# Heterogeneity of *Leishmania donovani* Parasites Complicates Diagnosis of Visceral Leishmaniasis: Comparison of Different Serological Tests in Three Endemic Regions

**DOI:** 10.1371/journal.pone.0116408

**Published:** 2015-03-03

**Authors:** Elfadil Abass, Cholho Kang, Franjo Martinkovic, Saul J. Semião-Santos, Shyam Sundar, Peter Walden, Renaud Piarroux, Abdallah el Harith, Michael Lohoff, Ulrich Steinhoff

**Affiliations:** 1 Institute for Medical Microbiology and Hygiene, University of Marburg, 35043 Marburg, Germany; 2 Biomedical Research Laboratory, Ahfad University for Women, P.O. Box 167, Omdurman, Sudan; 3 Faculty of Medical Laboratory Sciences, Sudan International University, Khartoum, Sudan; 4 Department for Parasitology and Parasitic Diseases with Clinic, Faculty of Veterinary Medicine, University of Zagreb, Heinzelova 55, 10000 Zagreb, Croatia; 5 Department of Nursing, University Tiradentes (UNIT), Campus Farolândia, CEP 49.032-490, Aracaju, Sergipe- Brazil; 6 Institute of Medical Sciences, Banaras Hindu University, Varanasi—221 005 UP, India; 7 Charité–Universitätsmedizin Berlin, Charitéplatz 1, 10117 Berlin, Germany; 8 UMR MD3 Aix-Marseille University, Marseille, France; Department of Medical Lab Technology, Fcaulty of Applied Medical Sciences, Taibah University, SAUDI ARABIA

## Abstract

Diagnostic tests for visceral leishmaniasis that are based on antigens of a single *Leishmania* strain can have low diagnostic performance in regions where heterologous parasites predominate. The aim of this study was to investigate and compare the performance of five serological tests, based on different *Leishmania* antigens, in three endemic countries for visceral leishmaniasis. A total number of 231 sera of symptomatic and asymptomatic cases and controls from three endemic regions of visceral leishmaniasis in East Sudan, North India and South France were evaluated by following serological tests: rKLO8- and rK39 ELISA, DAT (ITMA-DAT) and two rapid tests of rK39 (IT LEISH) and rKE16 (Signal-KA). Overall, rKLO8- and rK39 ELISA were most sensitive in immunocompetent patients from all endemic regions (96–100%) and the sensitivity was reduced to 81.8% in HIV co-infected patients from France. Sera of patients from India demonstrated significantly higher antibody responses to rKLO8 and rK39 compared with sera from Sudan (p<0.0001) and France (p<0.0037). Further, some Indian and Sudanese patients reacted better with rKLO8 than rK39. Sensitivity of DAT (ITMA-DAT) was high in Sudan (94%) and India (92.3%) but low in France being 88.5% and 54.5% for VL and VL/HIV patients, respectively. In contrast, rapid tests displayed high sensitivity only in patients from India (96.2%) but not Sudan (64–88%) and France (73.1–88.5% and 63.6–81.8% in VL and VL/HIV patients, respectively). While the sensitivity varied, all tests showed high specificity in Sudan (96.7–100%) and India (96.6%).Heterogeneity of *Leishmania* parasites which is common in many endemic regions complicates the diagnosis of visceral leishmaniasis. Therefore, tests based on homologous *Leishmania* antigens are required for particular endemic regions to detect cases which are difficult to be diagnosed with currently available tests.

## Introduction

Visceral leishmaniasis (VL) is a serious health problem in various countries and is caused by parasites belonging to the Leishmania donovani complex comprising two major subspecies, *L*. *infantum* mainly occurring in Latin America and Mediterranean region and *L*. *donovani* in East Africa and India [1]. However, it has been reported that both, *L*. *donovani* and *L*. *infantum* can be found in the same VL-endemic areas [2].

Strains of *L*. *donovani* in East Africa and *L*. *infantum* in the Mediterranean Basin are markedly heterogeneous and genetically different form Indian strains [3–5]. The parasites in East Africa are grouped into two genetically and geographically distinct populations, strains from South Ethiopia and Kenya and those from North Ethiopia and Sudan, corresponding to the distribution of their sandfly vectors, *Phlebotomus orientalis* in North Ethiopia and Sudan and *Phlebotomus martini* in South Ethiopia and Kenya [6].

The diagnosis of visceral leishmaniasis is difficult, both at laboratory and field level. Despite the availability of several diagnostic tests, none of these techniques alone is sufficient to identify all cases and results obtained by different tests varied in various regions. It has been suggested that varying diagnostic performance of these tests in the different VL regions is related to the origin of the test-antigen [7–8]. The Direct Agglutination Test (DAT), which detects agglutinating antibodies against surface antigens of *Leishmania*, is widely used for diagnosis of VL in several countries including Sudan [9–13]. The test uses *L*. *donovani* 1S promastigotes as antigen and is commercially available as freeze-dried antigen only from Institute of Tropical Medicine, Antwerpen and Royal Tropical Institute, Amsterdam.

Tests based on the recombinant protein K39 (rK39), a kinesin-related protein of *L*. *infantum* (Syn. *L*. *chagasi*), are reliable and are officially used for the national control strategy in countries such as India [14–15]. However, in some endemic countries like Sudan the sensitivity remains unsatisfactory, which as we showed, is related to low amounts of produced antibodies or the suboptimal test format[8].The low anti-*Leishmania* antibody responses of Sudanese VL patients has also been confirmed recently [16]. Another rapid test based on the recombinant kinesin-related protein KE16 (rKE16) of *L*. *donovani* from India, has a higher sensitivity in India compared to other regions [17–18]. We have recently cloned a homologous kinesin-related protein, rKLO8, of *L*. *donovani* strain from Sudan that consists of 294 amino acids containing the immunodominant repeats of 117bp [8]. The rKLO8 protein showed higher reactivity (OD values) with sera from Sudanese patients as compared to rK39, thus implying improved detection of patients with low antibody profile in Sudan.

The specifications for VL diagnostic tests vary among the different endemic regions. While malaria and HIV co-infections are common in some VL-endemic areas [19], asymptomatic infection poses further challenge for diagnosis and control of the disease in other areas [20–21]. In this study, we evaluated and compared the performance of five serological tests in well-characterized groups of patient and control sera collected from VL and non-VL subjects resided in three endemic regions in Eastern Sudan, Northern India and Southern France. The test panel included rKLO8- and rK39 ELISA, DAT (ITMA-DAT) and two rapid test kits of rK39 (IT LEISH) and rKE16 (Signal-KA). The possible reasons for variable performance in the different regions are discussed.

## Methods

### Serum samples

A total of 231 human serum samples of patients and controls were obtained from well-established serum collection banks (S1 Table). Sera were collected in three endemic regions of VL in Doka-East Sudan, Bihar-North India and Marseille-South France. They included 142 samples of VL and 11 of proven VL/HIV co-infection. Twenty-four sera were from symptomatic patients with unconfirmed VL diagnosis (VL suspects, VLS) from India and France. Twenty-five sera were from asymptomatic cases (ASC) from the same VL-endemic area in France. Control samples included 69 sera from healthy individuals without previous history of leishmaniasis or other diseases collected in a non-endemic area (non-endemic control, NEC) or from the same endemic area as VL (endemic controls, EC). The control sera included also sera from patients with malaria or toxoplasmosis.

### Selection criteria and characteristics of sera

Diagnosis of VL was established by detection of *Leishmania* amastigotes in Giemsa-stained aspirates of lymph nodes (Sudan), spleens (India) or bone marrows (France) and/or by culture in Novy, Nicolle and McNeal (NNN) medium (reference method). Patients with positive smear or culture results were considered confirmed VL. Diagnosis was done in the respective endemic area by experienced personals. Patients with typical symptoms but repeated negative smear or culture results were defined as unconfirmed VL (VLS). All sera of VLS from France have shown positive reactivity in ELISA and Western blot (WB) analysis using a soluble antigen of *L*. *infantum* (SLA) and were considered as highly suspected cases. Confirmed VL sera from France were subdivided based on results of HIV into HIV-negative with VL or HIV-positive with VL (VL/HIV). Testing of HIV infection was performed at Marseille teaching hospital using ELISA tests (ABBOTT diagnostics) and confirmed by immunoblot (BIO-RAD) and PCR (ABBOTT diagnostics). Sera of asymptomatic cases were selected based on results of *Leishmania* WB using the SLA of *L*. *infantum*. Sera of these patients reacted with the 14 and 16 kDa antigens, common in all *Leishmania* species [22]. Malaria and toxoplasmosis were diagnosed at the Biomedical Research Laboratory in Omdurman, Sudan or at the Department of Medicine at Banaras Hindu University, India.

### Ethical statement/consideration

The serum samples of this study have been used in former studies which have been approved and reviewed by institutional ethics committees in the respective countries as previously stated [8]. Serum samples have been collected on verbal consent of patients. Testing was anonymized and the scientific use of serum samples was approved by the local authority, Regierungspräsidium Gießen, Germany.

### Recombinant proteins

The recombinant protein KLO8 of *L*. *donovani* was produced and purified as recently described using M15 *E*. *coli* transformed with pQE41/KLO8 plasmid [8]. Expression was induced by adding 1 mM IPTG (Roth, Germany) for 4 hours and the recombinant protein was purified from the soluble fraction of bacterial lysates using nickel nitrile triacetic (Ni-NTA) columns (Qiagen GmbH, Germany).*L*. *chagasi* rK39 (rK39) was purchased from Rekom Biotech (S.L., Granada Spain). Both proteins, rKLO8 and rK39 antigens, were expressed as 6 x His-tagged fusion proteins and contain the same number of repeats (6 copy). Protein concentration was measured by Bradford test and aliquots were kept at -80°C until used.

### rKLO8- and rK39 ELISA

ELISA was performed using rKLO8 or rK39 as recently described [8]. The two recombinant proteins were used at a protein concentration of 5 ng/100μl and serum dilutions of 1:800. Optical densities (ODs) were measured at 450 nm using FLUOstar Omega ELISA microreader (BMG LABTECH). Individual sera were tested in duplicates and means were taken. Samples with inconsistent results were repeated. Known positive and negative sera were included as controls. A cut off value (0.12) for each recombinant protein was established as mean OD 450 plus 3 standard deviations (SD) of 30 sera from healthy controls.

### Direct agglutination test (ITMA-DAT)

ITMA-DAT kits (Lot 11D1B1) were purchased from the Institute of Tropical Medicine, Antwerp (ITMA, Belgium) and used to measure *Leishmania* agglutinating antibodies in serum samples. The antigen was reconstituted by adding 2.5 mL DAT buffer to each antigen vial and the test was performed in 96 V-shape microplates (Greiner Bio One, Germany) using full-scale titration according to manufacturer`s instructions. Sera were tested at two-fold serial dilutions, ranging from 1: 50 to 51200. The results were read after overnight incubation at R/T. The DAT titre is indicated as the highest dilution at which agglutination was still visible. Samples with titres of 1:≥3200 serum dilutions were considered as positive, whereas samples with titres of 1:800 and 1:1600 were considered as borderline.

### rK39 rapid test (IT LEISH)

Rapid tests based on rK39 (IT LEISH, REF 710124) were purchased from Bio Rad Laboratories (Marnes-la-Coquette, France) and stored at 4–8°C as recommended. IT LEISH is a dipstick rapid test for detection of anti-*Leishmania*-specific antibodies in human blood. The test device was pre-coated with *L*. *chagasi* (syn. *L*. *infantum*) recombinant antigen. During testing, patients’ specific antibodies captured by a conjugate (protein A-colloidal gold) react with the coated rK39 antigen on the membrane. This reaction is indicated by development of a specific colour. The test was performed and interpreted according to manufacturer`s instructions using 10 μL of serum samples. Tests were considered positive when purple control and test bands appeared. A single band in the control area indicates a negative result.

### rKE16 rapid test (Signal-KA)

“Signal KA flow through test kits” (Code No. 25993) were purchased from Span Diagnostics Ltd (Surat, India) and stored at 4–8°C until used. The test is based on detection of anti-*Leishmania* antibodies using rKE16 antigen of *L*. *donovani* [23]. The antigen is immobilised onto a permeable Nitrocellulose membrane and used to capture anti-*Leishmania* antibodies present in patients samples. Tests were performed and interpreted as described by the manufacturer. 50 μL serum samples were diluted 1:5 before being added into a nitrocellulose membrane of the test device. Tests were read within 10 minutes. Sera were considered positive if two magenta red dots, one for Control and the other for Test, appeared. One dot in the Control area indicates a negative result. The test was considered invalid if no dot appeared in the Control area.

### Study design

Anti-*Leishmania* antibodies response was measured serologically using two ELISA tests based on rKLO8 and rK39, DAT (ITMA) and two commercial rapid tests, based on rK39 (Bio Rad, France) and rKE16 (Span Diagnostics, India). These tests rely on antigens of different *Leishmania* parasites (S2 Table). DAT produced by ITMA is prepared from the Sudanese *L*. *donovani* 1S strain and rapid tests are based on recombinant proteins rK39 of *L*. *chagasi* or KE16 of *L*. *donovani*.

A panel of 231 sera obtained from Sudan, India and France were tested. Serum samples included true positive (confirmed VL with or without HIV, n = 113) and true negative (healthy and disease controls, n = 69) sera. In addition, 24 sera from symptomatic cases with unconfirmed diagnosis of VL (VLS) and 25 from asymptomatic cases from the same endemic areas were included. ELISA tests based on rKLO8 or rK39 were compared quantitatively to assess the performance for detection of anti-*Leishmania-* specific antibodies in the different regions. Cut off values were calculated for each protein by using 30 sera of healthy non-infected individuals. In the second part of the study, ELISA results were compared with those of DAT and two rapid tests. Sensitivity, specificity, positive predictive value (PPV) and negative predictive value (NPV) of each test were calculated and compared in the different endemic regions.

### Statistical analysis

The statistical analysis software GraphPad Prism (GraphPad Prism Inc., San Diego, Ca) was used to analyze the data. Significance was determined by unpaired Student's *t* test at 95% confidence intervals. Values of *P* < 0.05 were considered as significant. The validity of diagnostic tests was assessed by estimating sensitivity, specificity, PPV and NPV using standard formulas as follows: Sensitivity = True positives/ (True positives + False negatives) X 100%. Specificity = True negatives/ (True negatives + False positives) X 100%. PPV = True positives/ (True positives + False positives) X 100%. NPV = True negatives/ True negatives + False negatives) X 100%. Wilson’s score method was used to calculate confidence intervals [24].Sensitivity was calculated using sera of confirmed VL and VL/HIV groups whereas specificity was assessed combining results of all non-VL sera including healthy controls and patients with other diseases.

## Results

### ELISA

**Fig 1:**
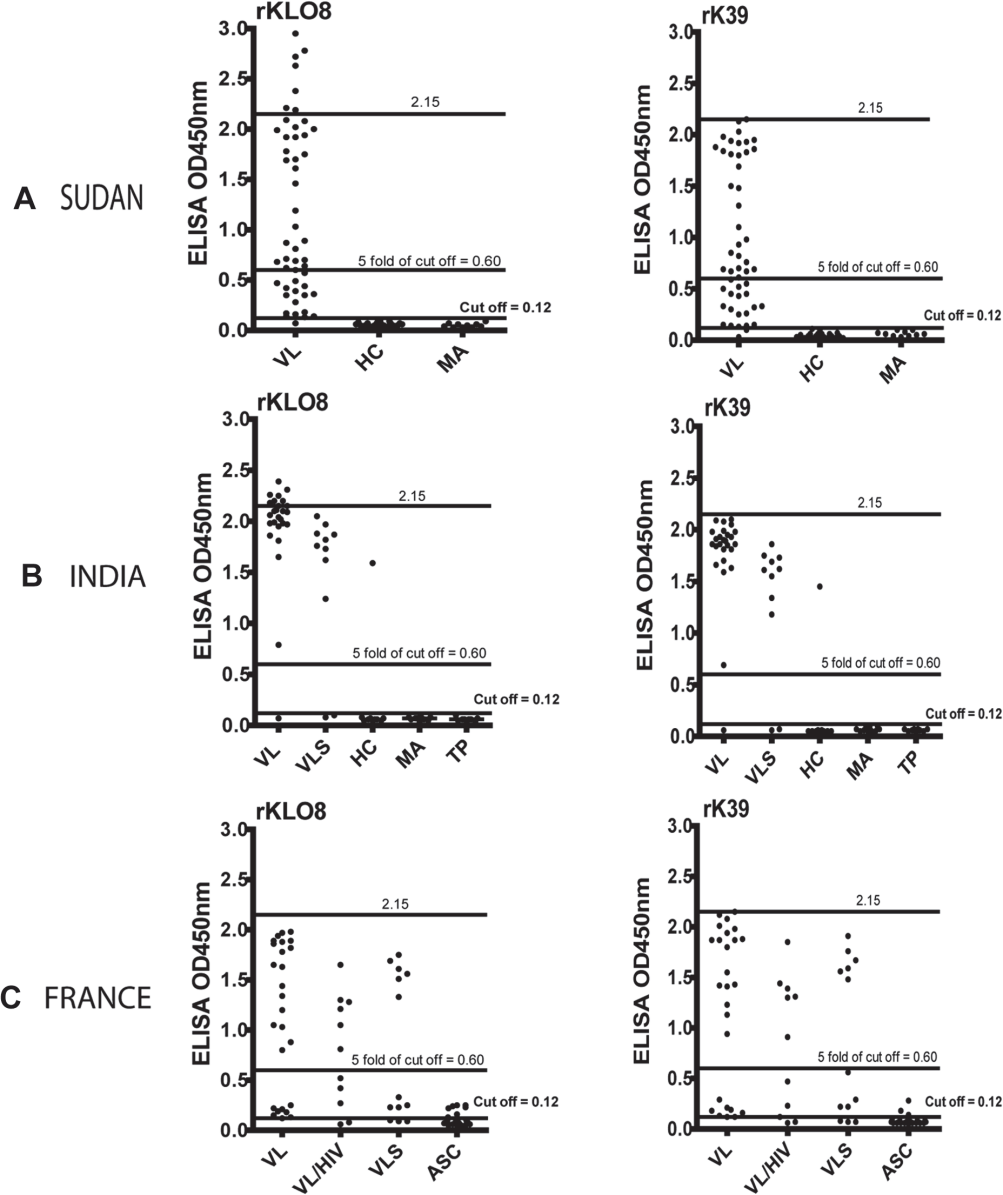
Comparison of anti-*Leishmania* antibody responses in rKLO8- and rK39 ELISA using patient and control sera from three endemic regions. Protein concentrations of 5ng/100μl rKLO8 or rK39 were tested with sera of patients and controls from Sudan (A), India (B) and France (C). Sera were tested at dilutions of 1:800. A cut off value of 0.12 was established as means + 3 SD of 30 healthy controls. VL, visceral leishmaniasis; VLS, VL suspects; HC, healthy control; MA, malaria; TP, toxoplasmosis; VL/HIV, VL and HIV, ASC, asymptomatic cases. The black horizontal lines represent cut off values. OD values of 0.6 (5 fold of cut off) and 2.15 are shown in dotted lines.

**Fig 2:**
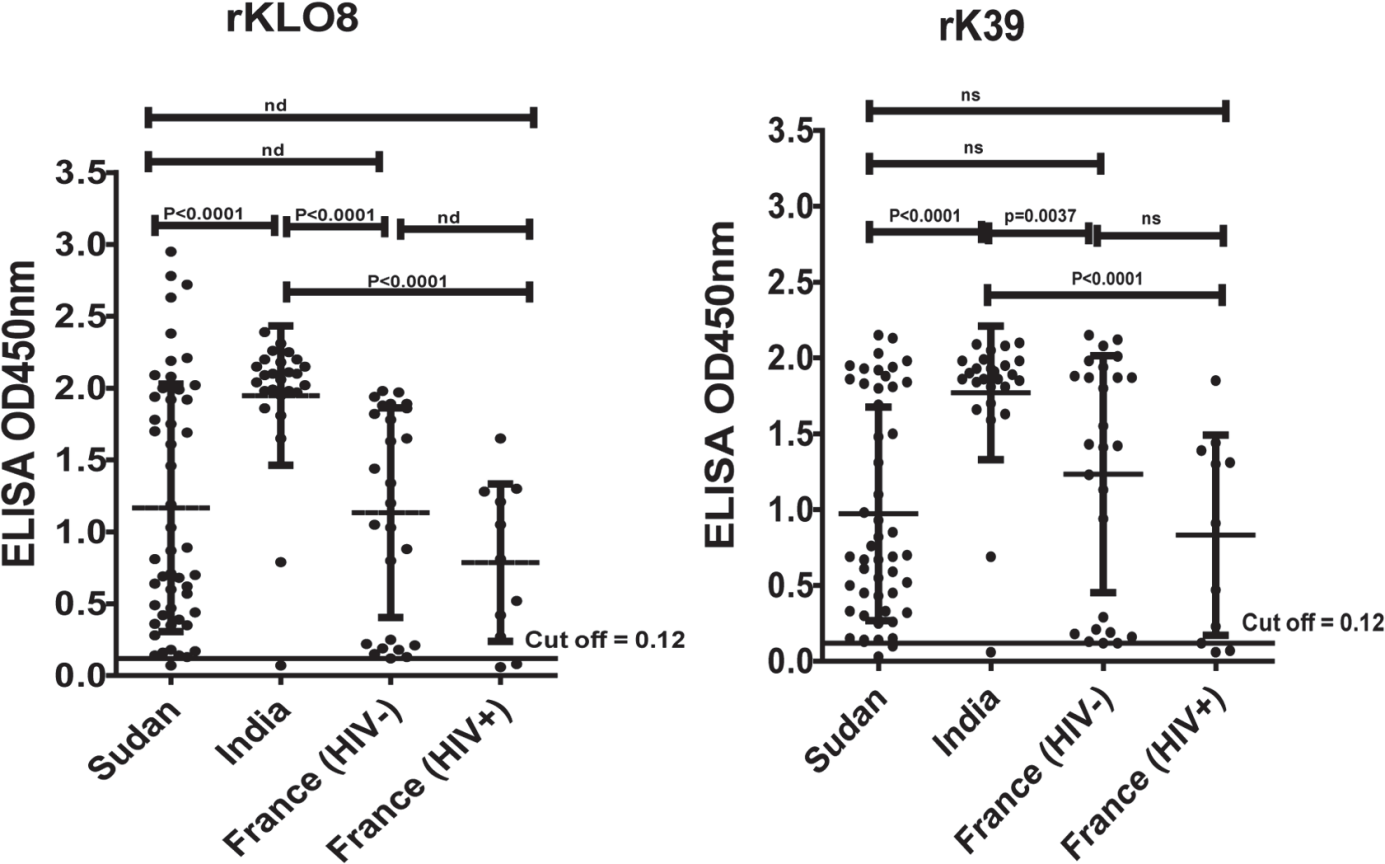
Reactivity of VL sera from different endemic regions in rKLO8- and rK39 ELISA. OD values of VL sera from Sudan (n = 50), India (n = 26) and France with negative (HIV-, n = 26) or positive (HIV+, n = 11) HIV were tested in rKLO8- or rK39 ELISA at dilutions of 1:800. Means ODs of VL sera from the different regions were compared. Dots represent values for individual sera and horizontal line represents cut-off values (0.12). Significance was determined by unpaired Student's *t* test at 95% confidence intervals and P-values < 0.05 indicate statistically significant differences; ns indicates no significant differences.

Antibody responses to the recombinant proteins rKLO8 and rK39 were compared by ELISA using sera of VL and controls from Sudan, India and France. Protein concentrations of 5 ng and serum dilutions of 1:800 were used as previously determined [8]. The results (Fig. 1) show that under these conditions, anti-*Leishmania* antibodies were detected in sera of VL from the 3 regions but at different levels of reactivity. In contrast to patients' sera from Sudan and France, most sera of VL from India showed similarly high OD values with both proteins. Out of 26 VL sera from India, 25 (96.2%) scored 5 fold the OD cut off value, both with rKLO8 and rK39 (Fig. 1A). These frequencies were only 33/50 (66%) and 30/50 (60%) for the VL patients from Sudan when tested on rKLO8 and rK39, respectively (Fig. 1B). Lower frequencies were also obtained when both proteins were tested with sera from French VL and VL/HIV patients being 18/26 (69.2%) and 6/11 (54.5%), respectively (Fig. 1C). Interestingly, sera of seven VL patients from Sudan and India showed higher ODs to rKLO8 than to rK39, reaching OD values of > 2.15 (Fig. 1A and 1B). Such values however were not observed with sera from French patients (Fig. 1C). In addition, sera of VL from India showed significantly higher reactivity (high OD values) to both rKLO8 and rK39 proteins as compared with patients from Sudan (p<0.0001) or France (p<0.0037) (Fig. 2). In contrast, no significant difference was found between Sudanese and French patients, nor between the VL and the VL/HIV co-infected groups from France.

The diagnostic performance of individual tests was calculated for each endemic region, as shown in Table 1. ELISA with both recombinant proteins showed a high diagnostic performance in the three endemic regions. The sensitivity was 98% and 96% in Sudan for rKLO8 and rK39, respectively, and similar in India (96.2%) and France (100%). Only one serum sample from a Sudanese VL patient showed a false-negative result with rKLO8 and only two with rK39. Sera from French HIV-coinfected (VL/HIV) patients revealed a markedly decreased sensitivity of 81.8%. Specificity values were equally high in the Sudan (100%) and India (96.6%). It is worth mentioning that one healthy individual from a VL-endemic area in India showed a strong positive signal (> 1.0) with both proteins (Fig. 1A). However, no cross-reactivity was observed with sera of malaria or toxoplasmosis patients. Accordingly, the PPV values of the two tests were similar in Sudan (100%) and India (96.2%). The NPV values were 97.6% and 95.2% for rKLO8 and rK39, respectively, in Sudan but the same for both proteins (96.6%) in India.

**Table 1:** Performance of five serological tests for diagnosis of visceral leishmaniasis in Sudan, India and France.

Region	Performance Index (%) at 95% CI		Serological tests				
			ELISA		DAT	Rapid test	
			rKLO8	rK39	(ITMA)	rK39 (BioRad)	rKE16 (Span)
**Sudan**	Sensitivity		98,0%	96.0%	94.0%	88.0%	64.0%
	Specificity		100%	100%	100%	97.5%	100%
	PPV		100%	100%	100%	97.8%	100%
	NPV		97.6%	95.2%	93.0%	86.7%	69.0%
**India**	Sensitivity		96.2%	96.2%	92.3%	96.2%	96.2%
	Specificity		96.6%	96.6%	96.6%	96.6%	96.6%
	PPV		96.2%	96.2%	96.0%	96.2%	96.2%
	NPV		96.6%	96.6%	96.3%	96.6%	96.6%
**France**	Sensitivity	VL (n = 26)	100%	100%	88.5%	88.5%	73.1%
		VL/HIV (n = 11)	81.8%	81.8%	54.5%	81.8%	63.6%

Abbreviation: VL/HIV, visceral leishmaniasis and human immunodeficiency virus; PPV, positive predictive value; NPV, negative predictive value; CI, confidence interval; DAT, direct agglutination test; ITMA, Institute of Tropical Medicine Antwerp. Specificity was calculated from negative controls including healthy individuals, and patients with malaria or toxoplasmosis. Sensitivity was calculated in parasitologically confirmed VL with HIV negative or positive results from France.

When testing rKLO8 and rK39 proteins in the VL suspect group, 9 out of 11 (81.8%) and 10 out of 13 (76.9%) serum samples from India and France were classified positive. Unexpectedly, rKLO8 (7/25; 28%) was more sensitive than rK39 (3/25; 12%) in detecting VL antibodies from asymptomatic patients in France. These results demonstrate the high sensitivity of rKLO8- and rK39- ELISA for detection of *Leishmania*-specific antibodies in immunocompetent VL subjects, with rKLO8 displaying higher reactivity in Sudan and India.

### DAT

Semiquantitative analysis of anti-*Leishmania* antibodies was performed with the direct agglutination test (DAT) with processed parasites of the *L*. *donovani* strain 1S. Antibody titres were determined and compared in sera of VL from the three regions. As shown in Table 2, patients from Sudan and India revealed higher antibody titres as compared with patients from France. DAT titres equal or higher than 1:51200 were scored in 86% and 92.3% of patients’ sera from Sudan and India, respectively. French patients showed relatively weaker DAT readings; strong titres (1:≥51200) were found in 57.7% of immunocompetent VL patients and in 36.4% of HIV-coinfected cases. The false-negative results (DAT titre ≤ 1600) were 11.5% for VL and 45.5% for VL/HIV co-infected patients. Such low titres were not observed in sera from Sudan and only in one serum of VL from India.

**Table 2:** Distribution of direct agglutination test (DAT) antibody titres in sera of visceral leishmaniasis patients from three endemic regions.

Origin of sera (no.)		Reciprocal DAT titres				
**Country**	**Number**	50–800	1600	3200–6400	12800–25600	≥51200
**Sudan**	(n = 50)	0 (0%)	3 (6%)	2 (4%)	2 (4%)	43 (86%)
**India**	(n = 26)	1 (3.8%)	1 (3.8%)	0 (0%)	0 (0%)	24 (92.3%)
**France**	HIV neg (n = 26)	3 (11.5%)	0 (0%)	3 (11.5%)	5 (19.2%)	15 (57.7%)
	HIV pos (n = 11)	5 (45.5%)	0 (0%)	1 (9.1%)	1 (9.1%)	4 (36.4%)

Values are number positive (%). DAT titres; 1: 50–1: 800, negative; 1:1600, marginal; 1:3200–1:6400, weak; 1:12800–1:25600, moderate; 1:≥51200, strong.

The sensitivity of DAT (Table 1) was high in Sudan (94%) and India (92.3%) as compared with France (88.5% in VL and 54.5% in VL/HIV sera, respectively). No cross-reactivity was observed in sera of the healthy or diseased controls from Sudan. One healthy subject from India was tested positive with a strong titre (1:>51200), resulting in 100% specificity in Sudan and 96.6% in India. Accordingly, the PPV was higher in Sudan (100%) than in India (96%) and the NPV values were similar in both countries (93.0%).

Within the VL suspect groups, 81.8% of the serum samples from India and 76.9% from France scored positive. In contrast, only one serum (4%) from the French asymptomatic patients (25) tested positive.

### Rapid tests

Qualitative detection of anti-*Leishmania* antibodies was assessed using two commercial rapid tests (RTs) based on rK39 and rKE16 antigens. Diagnostic performance for each test was calculated and compared in the three endemic regions (Table 1). Interestingly, only in India both tests showed high sensitivity (96.2% for both tests). Sensitivity was lower in Sudan and France, ranging from 88% to 88.5% (rK39 RT) and 64% to 73.1% (rKE16 RT), respectively. Sensitivity was further reduced when testing sera of VL/HIV co-infected patients from France; 81.8% for rK39 RT and 63.6% for rKE16 RT. The specificity of rKE16 RT was better than of rK39 RT (100% and 97.5%) in Sudan and was the same (96.6%) in India. A malaria patient from Sudan was tested positive with rK39 RT and a healthy subject from India cross reacted in both tests. The PPV values were therefore 97.8% for rK39 and 100% for rKE16 in the Sudan, but 96.2% for both tests in India. NPVs were the same in India (96.6%) and 86.7% and 69.0% in Sudan for rK39 RT and rKE16 RT, respectively.

In the VL suspects, 81.8% (9/11) of the serum samples from India were positive in both tests. In France, more cases were detected by the rapid test based on rK39 (10/13) than the one with rKE16 (7/13). With respect to asymptomatic cases, 3 sera tested positive in rK39 RT but were negative in the rKE16 RT.

## Discussion

VL can be caused by genetically distinct *Leishmania* parasites which due to their antigenic diversity trigger different host immune responses [25]. Diagnostic tests however are based on antigens of a single *Leishmania donovani* subspecies that might limit the diagnosis of the disease in some regions [18].

In this study, we compared the performance of rKLO8- and rK39 ELISA with the direct agglutination test (DAT-ITMA) and two rapid tests (IT LEISH and Signal KA) in three VL endemic regions where the disease is caused by different *L*. *donovani* subspecies. In general, performance of rKLO8- and rK39 ELISA was high in the three regions. The high sensitivity and specificity of rKLO8- and rK39 ELISA have been reported earlier by Chappuis et al [26] and by our group [8]. Of note, the strong antigenicity of rKLO8 and rK39 allowed detection of VL antibodies with high sensitivity even in immunocompromised VL patients, suffering from HIV co-infection whereas sensitivity of 81.8% was achieved. Unfortunately, geographical overlapping of VL and HIV occurs in many areas [27]. Thus we have to be aware that HIV co-infection, due to significantly reduced antibody generation, impedes the sensitivity of VL diagnosis [28–29].

Importantly, sera of VL from various countries showed different levels of reactivity towards rKLO8 and rK39 antigens, with a number of sera having reactivity just above the cut-off, and with only small variations in test performance could drop below the cut-off, thus creating a false-negative result. Nearly all tested sera of VL patients from India revealed higher antibody titres to both recombinant proteins compared with patients from Sudan and France (Fig. 2).Similar high antibody titres in Indian VL patients to *Leishmania* antigens have been reported earlier by others [16], which may be related to the high antigenic similarity of *L*. *donovani* parasites in India [5,7].

Our results confirmed the variable sensitivity of rapid tests based on rK39 and rKE16 in different endemic regions [18, 26]. Although both tests showed high specificity, their sensitivity is only satisfactory in Indian patients which have high antibody titres that may compensate for different reactivities of *Leishmania* antigens. Consequently, patients with low antibody titres as seen in Sudan and France can only be detected with low sensitivity. Interestingly, while the rK39 rapid test performs well in India but not in Sudan or France, the same antigen used in an ELISA format showed high sensitivity even in Sudan and France. This clearly demonstrates that not only the antibody titres of patients but also the test format determines sensitivity of VL diagnosis. In addition, the high sensitivity of DAT observed in India further confirms the impact of antibody levels on results of serological diagnosis. Beside its multivalent antigenicity in contrast to the single-antigen tests, use of DAT antigen of a Sudanese *L*. *donovani* strain (1S) might have contributed to its high sensitivity in Sudan despite the low antibody titres of this population. Similarly, in a previous study, sensitivity of DAT was favourably influenced by incorporating antigens from indigenous *L*. *donovani* strains [30].

Cross-reactivity of sera from malaria infected patients frequently limits the specificity of serological diagnostics for VL. Using high dilution of sera (1:800), we obtained 100% specificity in Sudan, thus eliminating the cross-reactivity against malaria which was observed when low serum dilutions of 1:100 were applied [8, 31, 32]. The healthy but seropositive control from India is most likely an asymptomatic case who was a housemate relative of a VL patient. During VL peak periods, the seroconversion rate has been reported to be high whereas the frequency of clinical disease in the same area remains low [33–35]. Thus, healthy individuals from these areas might include cases that are seropositive for *Leishmania* antigens such as healthy and asymptomatic cases. The fact that all of the herein studied cases from India with malaria and toxoplasmosis lack *Leishmania* seropositivity might be related to their distinct geographical distribution.

In East African endemic regions, VL and malaria share the same geographical distribution [19], thus co-infections of VL and malaria are common in several countries, including Sudan [36]. It has recently been shown that malaria co-infections manipulate immune responses of co-infected hosts[37].Beside other causes such as HIV or the nutritional status of patients[16,18],the low antibody titres observed in some VL patients could be attributed to aberrant host immune responses due to polyparasitisms. In these cases, combining results of different diagnostic tests using different *Leishmania* antigens will improve the diagnosis [8, 25].

In conclusion, our results show the complexity of VL diagnosis, as the available tests revealed variable performance in the three geographical regions and none of them reached absolute sensitivity and specificity. We therefore recommend that symptomatic patients with negative serology should be subjected to re-examination using different test systems and symptomatic patients with negative parasitology should be tested serologically. In addition, our data indicate that the performance of rapid VL diagnostic tests is influenced by the level of anti-*Leishmania* antibodies of patients and the source of *Leishmania* antigen.

## Supporting Information

S1 Checklist STARD checklist for reporting of studies of diagnostic accuracy. (DOC)

S1 Fig Flow diagram for heterogeneity of Leishmania Donovani parasites complicates diagnosis of visceral Leishmaniasis: comparison of three serological tests in three endemic regions. (TIF)

S1 Table Origin, source and number of serum samples. (DOCX)

S2 Table Source and origin of Leishmania antigens used in the serological tests. (DOCX)
