# Supplementary figures and images for: Heterogeneity of Leishmania donovani Parasites Complicates Diagnosis of Visceral Leishmaniasis: Comparison of Different Serological Tests in Three Endemic Regions
Source: PLoS One. 2015 Mar 3;10(3):e0116408. doi: 10.1371/journal.pone.0116408 (PMC4348478; doi:10.1371/journal.pone.0116408)

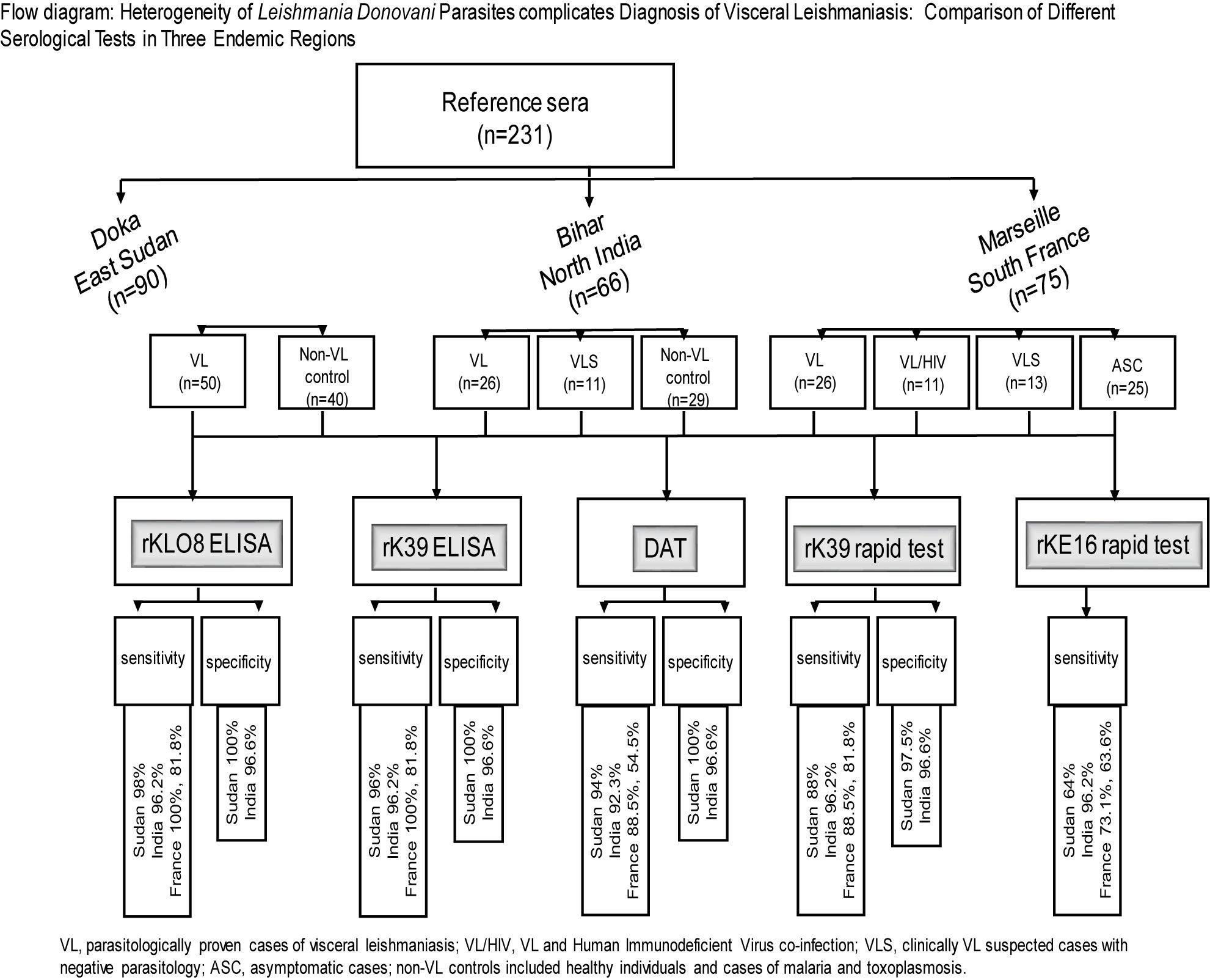

Supplement: S1 Fig — (TIF) [file pone.0116408.s002.tif]
