# Supplementary material for: Heterogeneity of Leishmania donovani Parasites Complicates Diagnosis of Visceral Leishmaniasis: Comparison of Different Serological Tests in Three Endemic Regions
Source: PLoS One. 2015 Mar 3;10(3):e0116408. doi: 10.1371/journal.pone.0116408 (PMC4348478; doi:10.1371/journal.pone.0116408)
Supplement: S1 Table — (DOCX) [file pone.0116408.s003.docx]

**S1 Table: Origin, source and number of serum samples.**

| Origin (no. of sera) | Source | Clinical condition (no. of sera) | |
| --- | --- | --- | --- |
| Doka, Eastern Sudan (n=90) | Biomedical Research Laboratory, Omdurman, Sudan | VL | (n=50) |
|  |  | Healthy | (n=30) |
|  |  | Malaria | (n=10) |
| Bihar, North India (n=66) | Department of Medicine,Banaras Hindu University, India | VL | (n=26) |
|  |  | VLS | (n=11) |
|  |  | Healthy | (n=10) |
|  |  | Toxoplasmosis | (n=9) |
|  |  | Malaria | (n=10) |
| Marseille, South France (n=75) | Laboratoire de parasitologie, Hôpital la Timone, Marseille, France | VL | (n=26) |
|  |  | VL/HIV | (n=11) |
|  |  | VLS | (n=13) |
|  |  | ASC | (n=25) |
| Total |  |  | 231 |
