# Supplementary material for: Heterogeneity of Leishmania donovani Parasites Complicates Diagnosis of Visceral Leishmaniasis: Comparison of Different Serological Tests in Three Endemic Regions
Source: PLoS One. 2015 Mar 3;10(3):e0116408. doi: 10.1371/journal.pone.0116408 (PMC4348478; doi:10.1371/journal.pone.0116408)
Supplement: S2 Table — (DOCX) [file pone.0116408.s004.docx]

**S2 Table: Source and origin of *Leishmania* antigens used in the serological tests.**

| **Antigen** | **Supplier** | **Parasite/origin** |  |
| --- | --- | --- | --- |
| rKLO8 This study *L. donovani* LO8/Sudan | | | |
| rK39 Rekom Biotech, Spain *L. infantum* LEM 589/France | | | |
| rKE16 Span Diagnostics, India *L. donovani* KE16/India | | | |
| DAT ITMA, Belgium *L. donovani* 1S/Sudan | | | |
